# Reciprocal Interaction of Wnt and RXR-α Pathways in Hepatocyte Development and Hepatocellular Carcinoma

**DOI:** 10.1371/journal.pone.0118480

**Published:** 2015-03-04

**Authors:** Jinyu Li, Maia Chanrion, Eric Sawey, Tim Wang, Edward Chow, Aaron Tward, Yi Su, Wen Xue, Robert Lucito, Lars Zender, Scott W. Lowe, J. Michael Bishop, Scott Powers

**Affiliations:** 1 Cancer Genome Center, Cold Spring Harbor Laboratory, Woodbury, NY 11740, United States of America; 2 Cancer Science Institute of Singapore, National University of Singapore, Singapore 117599, Singapore; 3 G. W. Hooper Foundation and Department of Microbiology and Immunology, University of California San Francisco, San Francisco, CA 94143, United States of America; 4 Cold Spring Harbor Laboratory, Cold Spring Harbor, NY 11724, United States of America; University of Medicine, Greifswald, Germany, GERMANY

## Abstract

Genomic analysis of human hepatocellular carcinoma (HCC) is potentially confounded by the differentiation state of the hepatic cell-of-origin. Here we integrated genomic analysis of mouse HCC (with defined cell-of-origin) along with normal development. We found a major shift in expression of Wnt and RXR-α pathway genes (up and down, respectively) coincident with the transition from hepatoblasts to hepatocytes. A combined Wnt and RXR-α gene signature categorized HCCs into two subtypes (high Wnt, low RXR-α and low Wnt, high RXR-α), which matched cell-of-origin in mouse models and the differentiation state of human HCC. Suppression of RXR-α levels in hepatocytes increased Wnt signaling and enhanced tumorigenicity, whereas ligand activation of RXR-α achieved the opposite. These results corroborate that there are two main HCC subtypes that correspond to the degree of hepatocyte differentation and that RXR-α, in part via Wnt signaling, plays a key functional role in the hepatocyte-like subtype and potentially could serve as a selective therapeutic target.

## Introduction

Most hepatocellular carcinomas (HCCs) develop after years of chronic liver inflammation during which time small nodular lesions develop from clonal expansion of hepatocytes and/or hepatic progenitor cells [1]. Recurrent genetic alterations that drive subsequent progression to malignancy include mutation of the β-catenin proto-oncogene *CTNNB1* [2], co-amplification of the neighboring proto-oncogenes *FGF19* and *CCND1* at 11q13.3 [3], or amplification or other genomic activation of the proto-oncogenes *MET* and *MYC* [4,5]. In addition, recurrent alterations affecting the tumor suppressor genes *TP53* and *DLC1* have been shown to promote HCC progression [6,7], and more recently, recurrent mutations affecting antioxidant response genes *NFE2L2* and *KEAP1* and histone methyltransferases genes of the *MLL* family have been found in multiple cohorts of hepatocellular carcinoma [8–10]. The only clinical trials of agents that target this set of oncogenic drivers in HCC are ones involving inhibitors of Met, although there is no biomarker guiding selection of patients in those trials [11].

Another possible avenue to matching HCC patients with specific treatments is through identification of molecular subtypes by transcriptional profiling. One study found subgroups with high Akt activation and proposed that these subtypes might respond well to inhibition of Akt [12]. However there are conflicting reports on the relationship between oncogenetic alterations and the molecular subtypes in HCC found by transcriptome profiling. In two studies, mutational activation of *CTNNB1* was found to be associated with Wnt pathway activation [12,13], whereas in a subsequent larger study, activating mutations were enriched in a class of differentiated hepatocyte-like tumors but Wnt pathway activation itself was associated with a class of tumors with wild-type *CTNNB1* [14]. Similarly, tumors with *TP53* mutations were associated with a proliferative class in one study but evenly distributed amongst all classes in a different study [12,13]. Such inconsistencies add uncertainty to the preclinical development of therapies that target specific pathways and to advancement of predictive biomarkers.

A major confounding factor in genomic analysis of HCC is that the tumor itself could arise from clonal expansion of a variety of starting normal cells along the hepatocyte lineage. In rodents, diethylnitrosamine (DEN) hepatocarcinogenesis oncogenically transforms mature hepatocytes, whereas the carcinogen furan activates bile duct progenitor cells giving rise to cholangiocellular carcinomas, and other carcinogenic regimens leading to HCC are thought to target either hepatoblast-like bipolar progenitor cells or the periductual stem cell [15]. There is also evidence based on comparative gene expression profiling of human tumors with rodent models that some HCC are derived from hepatic progenitor cells whereas others are not and instead retain differentiated features of hepatocytes [16].

Here we performed comparative genomic analysis of normal liver development and its relationship to both human hepatocellular (HCC) and mouse liver cancer models. Comparative oncogenomic approaches have previously been used to gain insight into the development of human HCC. Comparing mouse HCC models to their human counterparts led to the discovery that activating mutations of β-catenin co-occur with activation of the Met protein-tyrosine kinase, pinpointing a previously unappreciated cooperation between Wnt and Met signaling in HCC [17]. In another case, comparison of copy number alterations led to the identification of *YAP* and *IAP1* as chromosomally-linked cooperating oncogenes [18]. In this report we used comparative genomic analysis to address some unanswered questions regarding the relationships between recurrent genetic alterations, differentiation state, and pathway activation in HCC.

## Results

### Comparative oncogenomics of mouse and human HCC

**Fig 1:**
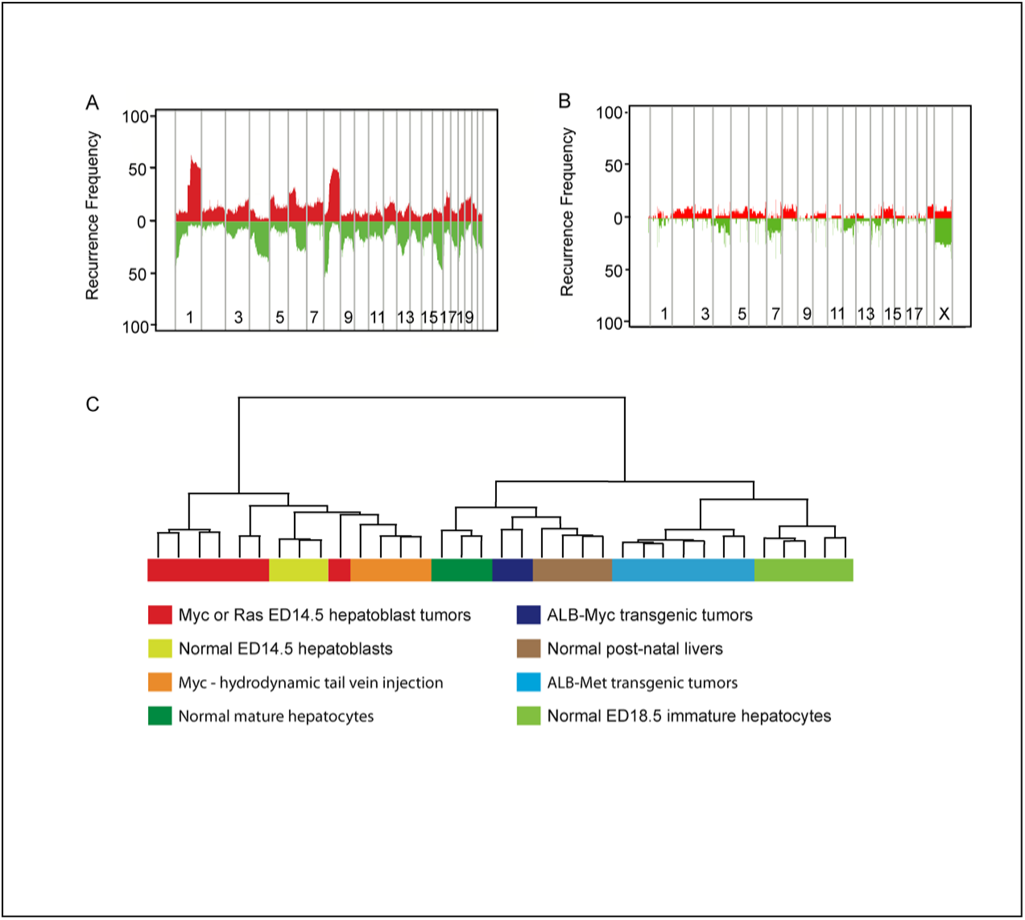
Comparative genomic analysis of human and mouse model HCC. (A) Frequency plots of copy number alterations in human HCC. Frequency of gains (red) and losses (green) as determined by ROMA array CGH analysis of 101 human HCC samples. (B) Frequency plots of copy number alterations in mouse model HCC as determined by ROMA array CGH analysis in 38 mouse model HCC samples. (C) Unsupervised hierarchical clustering of expression profiles of various mouse HCC tumors and normal stages of liver development. See S3 Table for a description of the different samples.

Previously we used array CGH (ROMA) to discover focal amplicons in eight tumors derived from two different hepatoblast transplantable mouse HCC models. These focal amplicons included a *Myc* amplicon found in *RAS*/*p53* ^-/-^ hepatoblast tumors and a *YAP1/BIRC2* amplicon found in several independent *MYC*/*p53* ^-/-^ hepatoblast tumors [18]. Here we profiled copy number alterations in an additional thirty mouse HCC tumors, including ten additional transplantable hepatoblast-derived tumors and twenty tumors derived from transgenic mice. The transplantable hepatoblast-derived tumors were generated from hepatoblasts isolated from either *p53* ^-/-^ or *Cdkn2a* ^-/-^ mice that by retroviral transfection given a second oncogenic hit (overexpression of *MYC*, activated *RAS*, or activated *RHO*, or shRNA-silencing of *DLC1*). The twenty tumors from transgenic mice included ten expressing human *MET* specifically in hepatocytes under the control of doxycycline [17], and ten from mice expressing the human *MYC* gene driven by the albumin promoter. The most frequently observed focal copy number alteration was the *YAP1/BIRC2* amplicon (9qA1), observed in five of the transplantable hepatoblast-derived tumors. Other focal amplicons were observed only once and only in the transplantable hepatoblast-derived tumors, and included the *MYC* and *RNF19* amplicons previously reported, and amplicons containing *MTTP* (3qG3), *PKN2* (3qH1), *FGFR2* (7qF3), *STK32C* (7qF4), and *SCN3B* (9qA5) (S1 Table). We did not observe any focal deletions and furthermore we observed neither focal nor broad copy number alterations in any of the twenty tumors isolated from transgenic models. There were relatively few broad copy number gains or losses in tumors arising from the transplantable mouse tumors, with broad losses involving chromosomes 4, 7, and 12 and broad gains involving chromosomes 2, 3, 5, 6, 8, 15, and 19 observed in 11% to 28% of the 18 transplantable mouse tumors, compared to the more frequent broad copy number alterations observed in human HCC (Fig. 1).

Since the low frequency of DNA copy number alterations in mouse HCC tumors did not lend itself to a comprehensive comparison with copy number alterations in human HCCs, we turned our attention to comparison of gene expression alterations. We first focused on gene expression alterations in mouse tumors, as their initiating oncogenic lesions are known and they are less heterogeneous than human HCC. Only sixteen of the thirty mouse tumors analyzed for DNA copy number alterations yielded RNA suitable for genome-wide analysis, so we added an additional four mouse hepatoblastomas that were generated by hydrodynamic tail-vein injection of an inducible *MYC* expression plasmid [19]. Thus we analyzed three different types of mouse HCC models driven by *MYC* overexpression: the transplantable hepatoblast model where p53^-/-^ hepatoblasts were retrovirally transduced with a *MYC* overexpression plasmid, a hepatoblastoma model where tail-vein injection is used to delivery a TET-inducible *MYC* transgene directly into liver cells (the presumed cell-of-origin was deduced from histological analysis of the resultant tumors[4]), and a classic transgenic model where the hepatocyte-specific *ALB* promoter is used to drive *MYC* expression. We analyzed in parallel normal mouse liver tissue taken from different stages of development. By unsupervised cluster analysis of the transcriptomes of these samples that there were two major clusters (Fig. 1). These two clusters were comprised of samples with similar stages of liver differentiation (Fig. 1 and S1 Fig.). Tumors that either were known[18] or suspected[4] to originate from hepatoblasts, regardless of whether the initiating oncogene was *MYC* or *RAS*, grouped together with hepatoblast-enriched fetal liver from embryonic day 14.5, whereas other mouse tumors initiated by *MYC* or *MET* but that arise from hepatocytes grouped together with normal liver at later stages of development (Fig. 1 and S1 Fig.; a complete listing describing the mouse tumors is found in S3 Table).

### Wnt/β-catenin pathway genes and retinoic acid receptor X (RXR-α) pathway genes are major features of the two subtypes of mouse HCC

**Fig 2:**
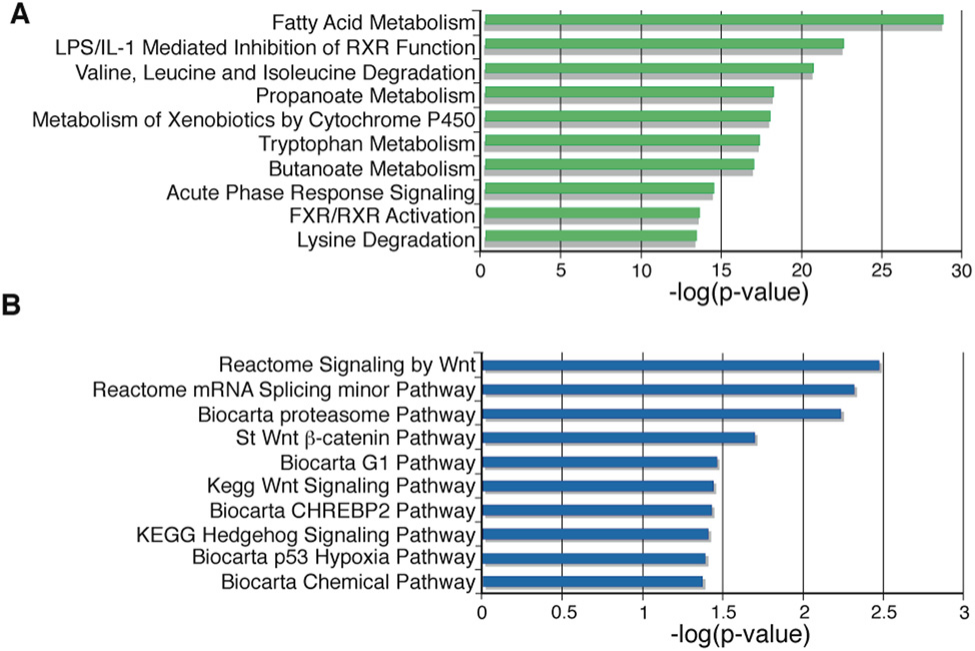
Pathways that distinguish the two major clusters of mouse HCC. Top ten most significantly different pathways based on gene expression of the two major clusters of mouse HCC and normal liver samples determined by GSEA (A) and Ingenuity (B).

We then used pathway analysis of the transcriptome data to determine what pathways were most significantly altered between the two groups. We used two commonly used computational pathway tools (Ingenuity and GSEA) to determine which pathways (as defined by gene sets) showed statistically significant, concordant expression differences between the two groups. Two of the ten most significantly altered pathways pinpointed by Ingenuity involved RXR-α, a nuclear hormone receptor which forms heterodimers with several other nuclear hormone receptors including RARs, LXRs, FXR, and PPAR-□ [20] (Fig. 2A). Three of the top ten most significantly altered pathways pinpointed by GSEA involved Wnt/β-catenin signaling (Fig. 2B). Both of these pathways have established roles in liver development [21] [22].

We then tested whether, instead of the entire transcriptome, a much smaller gene set defined by RXR-α and Wnt signaling could be used to similarly categorize mouse model HCCs. We formed the gene signature by combining Wnt signaling genes (Reactome [23]) with RXR-α genes (Ingenuity Systems Inc.) (S2 Table). As with the clustering based on all genes, clustering with the combined pathway gene signature partitioned mouse model HCCs into two subtypes (high Wnt, low RXR-α and low Wnt, high RXR-α) that corresponded to the cell-of-origin (S2 Fig.).

### Reciprocal relationship between RXR-α and Wnt pathways in normal liver development

**Fig 3:**
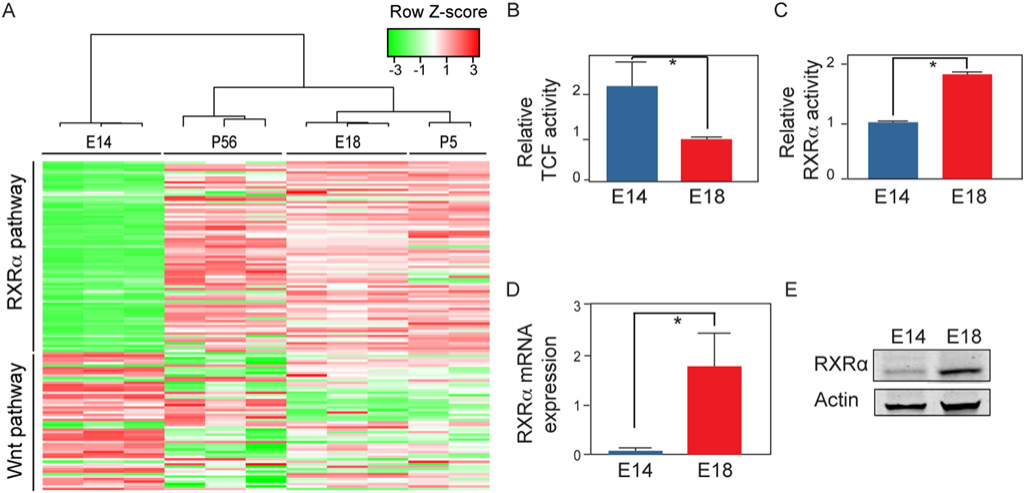
Gene expression and biochemical changes in RXR-α and Wnt pathways during liver development. (A) Clustering of RXR-α and Wnt pathway gene expression profiles of normal mouse liver samples taken from four different periods of development. (B) Reporter assays for Wnt and RXR pathways comparing hepatoblasts (E14) to immature hepatocytes (E18). Both TCF (p = 0.016) and (C) RXR-α activities (p = 0.021) were significantly different (D) RNA and (E) protein expression of RXR-α in hepatoblasts compared to immature hepatocytes.

We then examined the relationship between different stages of normal liver development and the transcriptional targets of RXR-α and Wnt signaling. We used unsupervised clustering to find patterns in the expression levels of these transcriptional targets from liver samples isolated at embryonic days E14.5 and E18.5 as well as later postnatal stages (P5 and P56). This analysis revealed a major shift in the expression of the transcriptional targets of both pathways occurring between E14.5 and E18.5, with transcriptional targets for Wnt signaling upregulated at E14.5 and down regulated in E18.5 and the opposite result for transcriptional targets of RXR-α signaling (Fig. 3A). The pattern established by E18.5 was maintained during later stages (Fig. 3A). In an independent transcriptome dataset covering several different stages of liver development, there was a similar shift involving both Wnt and RXR-α pathway transcriptional targets occurring between E16 and birth (S3 Fig.). Together, these results demonstrate that a major shift in the activity of both the Wnt and the RXR-α pathways occurs between E16 and E18.5.

To confirm these findings, we used transcriptional-reporter assays and found that there was a 2-fold down-regulation of Wnt signaling in E18.5 immature hepatocytes relative to E14.5 hepatoblasts (Fig. 3B), concurrent with a near 2-fold reduction in RXR-α signaling (Fig. 3C). We also determined that there was a major increase in the levels of RXR-α mRNA and protein during the transition from hepatoblasts to immature hepatocytes (Fig. 3D and 3E).

### Combined Wnt and RXR-α gene signature classifies human HCC into two subtypes

**Fig 4:**
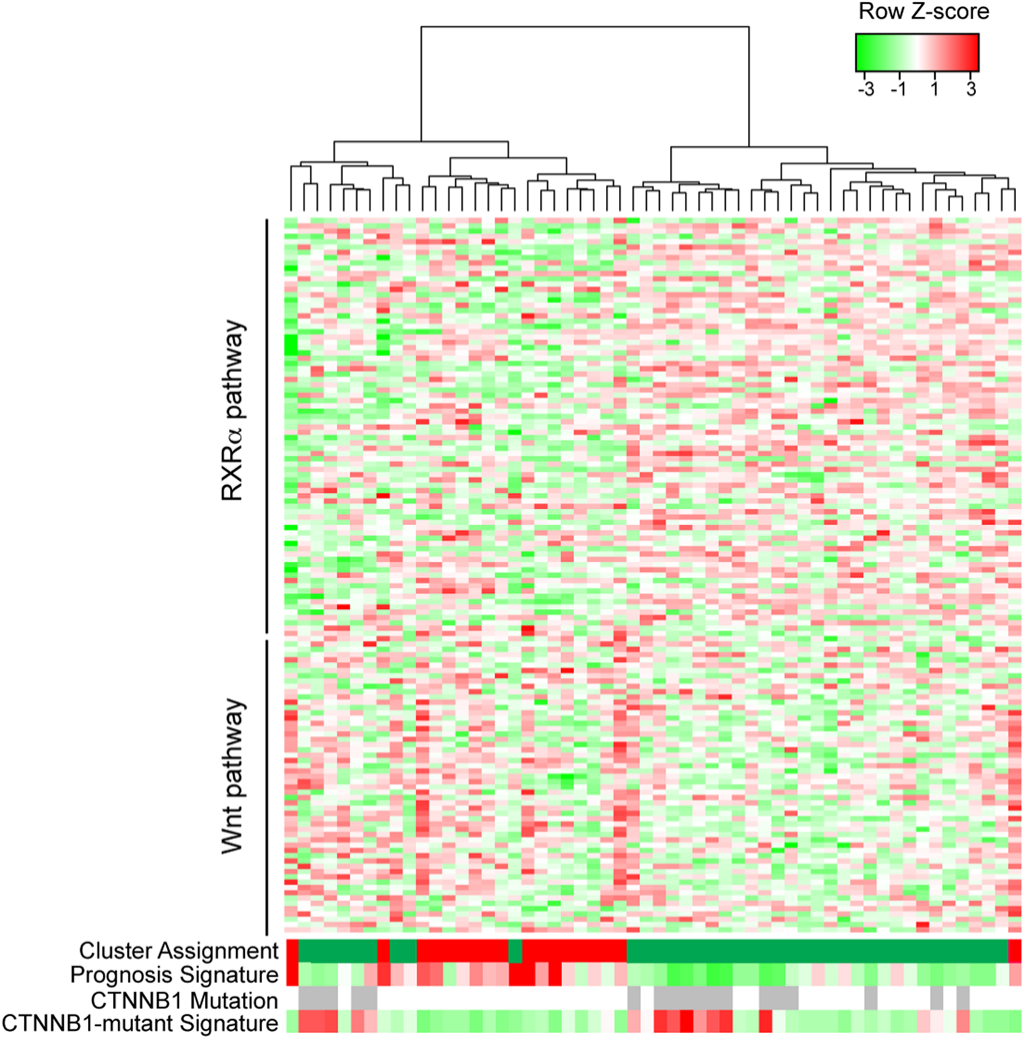
Classification of human HCC based on expression of RXR-α and Wnt pathway genes. HCC samples in the Boyault dataset[12] were clustered based on expression of 138 RXR-α and Wnt pathway genes. Beneath the heatmap are four rows, showing for each HCC sample (1) cluster assignment to the two major groups found by unsupervised clustering of all genes (2) relative prognosis based on the 65-gene signature of Kim et al.[24], ranging from red = poor, white = neutral, green = good; (3) grey bars indicate activating mutation in *CTNNB1*; (4) average expression of the 5 genes known to be overexpressed in *CTNNB1*-mutant HCC cells; red = expression, green = less expression of the 5-gene signature associated with *CTNNB1* mutation[12].

We then used the combined Wnt and RXR-α pathway gene signature to analyze human HCC. Unsupervised clustering with this signature using the Boyault et al. dataset [12] revealed two major classes, one that exhibited higher expression of Wnt transcriptional targets and lower expression of RXR-α transcriptional targets (Wnt high, RXR-α low), and the opposing group (Wnt low, RXR-α high) (Fig. 4), similar to the classification we observed with the signature in mouse HCC (S2 Fig.). Additionally, the clusters identified by the combined Wnt and RXR-α pathway gene signature overlapped significantly with the two major clusters formed by analysis of the entire transcriptome (Fig. 4). Quantification of a 65 gene-based risk score classifier that predicts overall survival in hepatocellular carcinoma[24] revealed that the Wnt high, RXR-α low group has much lower predicted rate of survival than the Wnt low, RXR-α high group (Fig. 4). Analogous to what we observed in mouse HCC, the classification of human HCC based on the combined Wnt and RXR-α pathway gene signature was highly reflective of the two major groups found by hierarchical clustering based on the entire transcriptome (Fig. 4).

Seemingly paradoxical but similar to the study of Hoshida et al. [14], we observed less *CTNNB1* mutations in the group associated with Wnt pathway activation than we did with the group associated with lower Wnt pathway activation (Fig. 4). However, there is a high degree of cell-context dependency in the transcriptional targets of β-catenin activation [25] and as would be expected, *CTNNB1*-mutant HCC showed a strong tendency to overexpress the five genes previously found to be selectively overexpressed in *CTNNB1*-mutant HCC (Fig. 4) [26–28]. The discrepancy between this 5-gene signature and the Wnt pathway signature is addressed in the discussion.

The combined Wnt and RXR-α pathway gene signature also classified human HCC expression profiles in two other datasets into two basic groups (Wnt high, RXR-α low and Wnt low, RXR-α high) that reflected both prognosis and the grouping based on whole transcriptome analysis. This included the Wurmbach et al. dataset[25] of HCC and non-malignant liver samples (S4 Fig.) and the Kim et al. dataset[24] (S5 Fig.). Thus the combined Wnt and RXR-α pathway gene signature performs consistently across three distinct human HCC datasets: it defines two major clusters that correspond closely to the two major clusters formed by considering the entire transcriptome, and it also corresponds closely to the poor prognosis signature generated by Kim et al. [24].

### Modulating RXR-α activity alters β-catenin signaling and affects tumorigenicity

**Fig 5:**
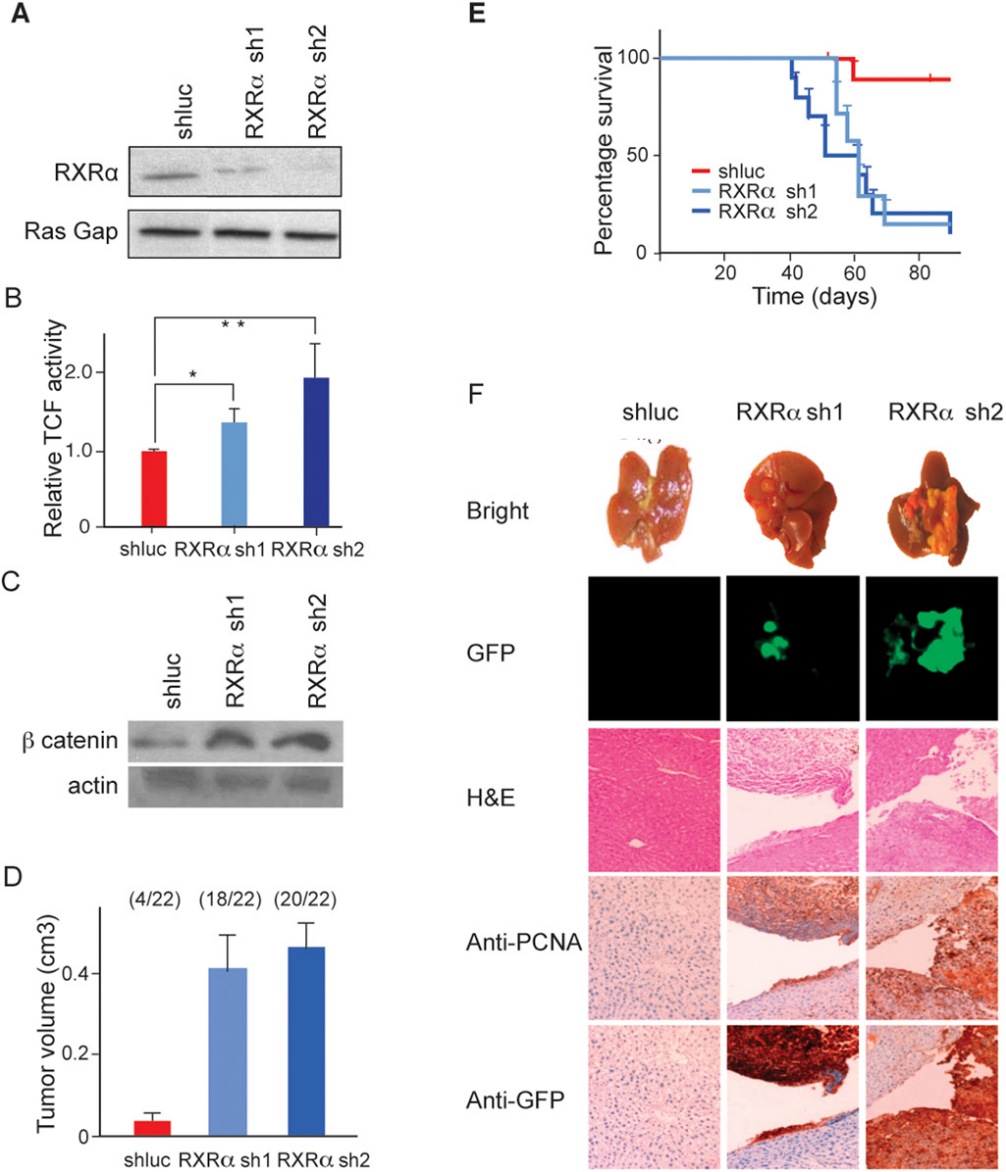
Effects of lowering RXR-α protein levels in hepatocytes on Wnt signaling and tumorigenicity. (A) Validation of two independent shRNAs for their ability to lower RXR-α protein levels in hepatoctyes as determined by immunoblotting using Ras Gap protein expression as a loading control and shRNA directed against luciferase as a vector control. B) Tumor growth following subcutaneous injection in nude mice of MYC/p53^-/-^; E18 hepatocytes infected with either shluc (red column), RXRA sh1 (purple column), or RXRA sh2 (blue column). Error bars indicate standard deviations. Tumor incidence is noted above columns for each condition. **C**) Survival curves of nude mice after intrasplenic injections of *MYC/p53^-/-^*; E18 hepatocytes transfected with either shluc (red line), or RXRA sh1 (purple line), or RXRA sh2 (line), n = 10 injections. D) Images of livers taken from mice following transplantation of *MYC/p53^-/-^* E18 hepatocytes transfected with shluc, RXRA sh1, or RXRA sh2. The five panels are from left to right, intact livers, GFP-imaging of livers, hematoxylin and eosin staining of liver tissue sections, PCNA immunohistochemical staining, GFP immunohistochemical staining. Size bar = 200 μm.

We then tested whether reducing hepatocyte RXR-α levels affected either Wnt signaling or tumorigenicity. We screened for two independent shRNAs that were both effective at reducing the level of RXR-α protein in hepatocytes (Fig. 5A). Both of these shRNAs increased Wnt activity (Fig. 5B) and elevated β-catenin levels (Fig. 5C). Additionally, these shRNAs induced *Myc/p53^-/-^* hepatocytes to form large tumors when injected subcutaneously (Fig. 5D) and led to rapid liver cancer formation *in situ* (Fig. 5E). Histological examination of the tumors formed *in situ* revealed that they were composed of a population of proliferative, undifferentiated cells that resembled human HCC (Fig. 5F).

**Fig 6:**
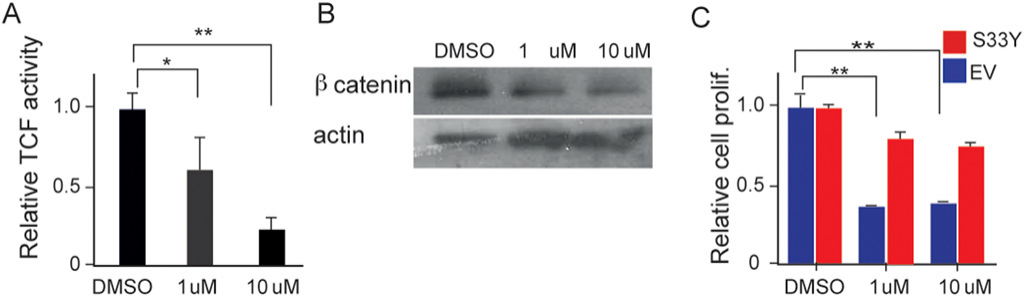
9-cis retinoic acid activation of RXR-α suppresses β-catenin signaling. (A) Effect of 9-cis retinoic acid on Wnt signaling activity in Huh-7 and HLE cell lines as measured using a TCF transcriptional reporter. Labeling as in Panel B. (* = p < 0.05; ** = p < 0.001). (B) Effect of 9-cis retinoic acid on β-catenin protein levels in Huh-7 cells. (C) Effect of 9-cis retinoic acid on clonogenic growth of Huh-7 cells transfected with either mutationally activated *CTNNB1* (S33Y; red column) or empty vector (blue column) ((p = 0.0037 and 0.0041). In all panels, error bars indicate standard deviations.

We then looked at the effect of stimulating RXR-α with its ligand 9-cis retinoic acid. To determine stimulating RXR-α might suppress Wnt signaling, we tested the effects of 9-cis retinoic acid on a Wnt-pathway transcriptional reporter in the hepatocyte-like cell line Huh7. Wnt activation was reduced approximately 50% with 1 μM 9-cis retinoic acid and reduced 80% at higher dosage (Fig. 6A). These levels of 9-cis retinoic acid also reduced β-catenin protein levels (Fig. 6B). Expression of mutationally activated *CTNNB1* significantly reduced the ability of 9-cis retinoic acid to suppress clonogenic growth (Fig. 6C).

## Discussion

Despite years of genomic studies aimed at molecular classification of human HCC, there is still concern about lack of overlap between different studies[29]. A computationally oriented approach to resolve discrepancies provided a meta-analysis of several studies with the conclusion that there were three basic subgroups [14]. Here we took a biologically-oriented approach based on mouse models of HCC and normal liver development that instead indicates two basic subtypes of both mouse and human HCC that correspond to either a hepatoblast or hepatocyte differentiated state, corroborating earlier findings of Thorgeirsson’s group [16,30]. Moreover, we found that in mouse models of HCC that the cell-of-origin plays the dominant role in overall transcriptional patterns irrespective of the initiating oncogene. This sheds light on why it has been difficult to consistently explain the heterogeneity of human HCC based on the underlying genetic alterations [29].

We found that expression of RXR-α and Wnt pathway genes are key features of the transcriptional changes that distinguish the two subtypes of mouse model and human HCC. Both of these pathways play key roles in hepatocyte development. Deletion of β-catenin in mouse hepatoblasts prevents their maturation, expansion, and survival [21]. Similarly, deletion of RXR-α has been shown to dramatically delay liver differentiation [22]. In our study we found an inverse relationship between Wnt and RXR-α pathway transcription, with a major reduction in Wnt target genes coincident with a major increase is RXR-α target genes occurring between E16 and E18.5, a developmental period where the majority of hepatoblasts acquire an epithelial morphology and become arranged into epithelial sheets [31].

Hepatocellular carcinomas that have de-differentiated into hepatoblasts appear to have intrinsically higher Wnt signaling. The relatively low Wnt signaling activity of hepatocytes may form a tumor suppressor barrier, providing the selective pressure underlying the high frequency of activating *CTNNB1* mutations in this subtype. In previous studies where mutational activation of *CTNNB1* was found to be associated with Wnt pathway activation [12,13] the genes used to define Wnt pathway activation were genes that had been specifically found to be upregulated in *CTNNB1*-mutant HCCs, rather than standard Wnt signaling genes used in our study (e.g. *BTRC*, [32]. There can be considerable tissue-specificity and temporal-restrictiveness in the transcriptional targets of Wnt pathway activation and it is very likely that a significant proportion of Wnt signaling target genes differ depending on the differentiated state of the hepatocytes [33].

In this study we showed that RXR-α has tumor suppressive function in hepatocytes and that reducing its expression increases β-catenin levels and signaling, whereas increasing RXR-α function by ligand activation lowered β-catenin levels and signaling. These findings are in agreement with a previous study using other cancer cell types where ligand activation of RXR-α was shown to enhance degradation of β-catenin independently of the APC-proteasomal degradation pathway [34]. However, RXR-α does not appear to be a frequent target for genetic or epigenetic inactivation in HCC [35], which may reflect a requirement of residual level of its function for tumor maintenance. In our study, shRNA-mediated suppression of RXR-α reduced but did not eliminate the protein.

## Methods

### Ethics

This study was approved by Cold Spring Harbor Laboratory’s Institutional Animal Care and Use Committee (IACUC). The Cold Spring Harbor Laboratory animal facilities are fully accredited by the American Association for Accreditation of Laboratory Animal Care. Animals are maintained in accordance with the applicable portions of the Animal Welfare Act and the DHHS “Guide for the Care and Use of Laboratory Animals”. All mice were monitored closely for pain and suffering as a result of tumor burden and sacrificed prior to or immediately upon observation of discomfort. Specific criteria for euthanasia was as follows: 1) further observation is no longer necessary for the purposes of the study (i.e. tumors have appeared and grown large enough for isolation of DNA or cells) OR 2) upon observation of substantial weight loss, abnormalities with movement or breathing, excessive lethargy or tremors, or upon the advice of the facility veterinarian. Upon a decision to sacrifice, tumor tissue was recovered from the animal for genetic and histological analysis. Mice were euthanised by carbon dioxide asphyxiation as recommended by the 2000 Report of the AVMA Panel on Euthanasia, JAVMA, vol. 218, No. 5, March 1, 2001.

### Cell line

HCC cell line Huh-7 was obtained from ATCC and grown in RPMI medium with 10% FBS and 1% Pen-Strep.

### Statistical analysis

All experiments with animals or cell lines were done in triplicate or quadruplicate. Student’s t-test was used to access significance of differences in two populations and error bars indicate the standard error of the means.

### Genomic analysis

Total RNA from whole livers taken at different developmental timepoints (embryonic day 14, embryonic day 18, post-natal day 5 and post-natal day 56) along with hepatoblasts isolated from E14 livers and immature hepatocytes isolated from E18 livers was extracted and purified using the Qiagen RNeasy Mini Kit. RNA purity and integrity were assayed by the Bioanalyser 2100 (Agilent Technologies). For each sample, 2 μg of total RNA was reverse transcribed and amplified by using an RNA amplification kit from Ambion. Fifteen micrograms of amplified RNA were labeled by direct chemical coupling to the Cy5 NHS ester (Amersham Biosciences). Normal adult mouse liver (Agilent) was used as control and Cy3 labeled. Labeled RNAs were purified, fragmented, and used as probes to hybridize microarrays. Gene expression profiling was done with the 4x44k mouse Agilent platform. Expression profiling of the 23 human HCC samples was previously described^21^.

### Computational analysis

Microarray data for normal liver samples used in this study was normalized and analyzed with limma R package. Gene expression datasets from different platforms were combined after scaling by setting the mean to zero and the Standard deviation to 1. Significance Analysis of Microarrays (SAM) was used to identify differentially expressed genes between the two clusters shown in Fig. 2. Pathway analysis of differentially expressed genes was performed using Ingenuity Pathway Analysis (Ingenuity Systems, Redwood City, CA) and GSEA. GSEA analysis was performed using software downloaded from Broad Institute ((www.broadinstitute.org/gsea/<http://www.broadinstitute.org/gsea/>). RXR-α pathway genes were obtained from Ingenuity Gene Sets, and Wnt pathway genes were obtained from Reactome (www.reactome.org<http://www.reactome.org/>). Clustering and heatmaps were performed using the R software.

### Cloning

RXRα shRNA constructs in polIII-promoter based pSM2c vectors were obtained from Open Biosystems (Huntsville, AL). shRNA inserts were subcloned using EcoRI and XhoI restriction sites into the *mir30*-cassette of the polII-promoter based expression vector (MSCV)-LTRmiR30-puro^22^. The mouse RXRα gene (kindly obtained from Dr. Chambon, IGBMC) was subcloned from the pSG5 vector into the pcDNA3.1 vector using EcoRI. It was then partially digested with BamHI and subsequently cut with XhoI to be cloned into the pMSCV-hygro vector (Clontech, Palo Alto, CA). Human RXRα cDNA was PCR amplified from the pBABE-hygro human RXRα (plasmid 11440 from Addgene) using primers with engineered BglII and XhoI restriction site sequences. The product was then cloned into the BglII and XhoI sites of the MSCV-hygro vector. MSCV-murine-Myc-IRES-GFP vector was kindly obtained from Dr. Scott Lowe’s laboratory. Bing packaging cells were plated on 60 mm dishes prior to being transfected with 2μg of helper plasmid and 4μg of target DNA (cloned into an MSCV vector) using the calcium phosphate method (E1200, Promega). After 48 hours, cells were infected using filtered viral supernatant supplemented with 8 μg/ml polybrene. The infection procedure was repeated three times every 8 hours. 24 hours after the latest infection, infected cells were selected using puromycin or hygromycin at 3 μg/ml and 500μg/ml respectively.

Liver hepatoblast/hepatocyte isolation and immortalization

Day 14 and 18 liver embryos of C57BL/6 mice were harvested and cultivated modified from a protocol described previously^12^. Briefly, after dispase treatment for 30 minutes at 37°C, filtered cells were E-Cadherin immunopurified (Calbiochem, 205603) using the MACS technology (Miltenyi Biotec). Collected cells were plated on gelatin-coated plates with gamma-irradiated NIH3T3 feeder layer cells in serum free HGM medium^23^. To immortalize p53^-/-^ harvested hepatoblasts, kindly obtained from Dr. Lowe, cells were retrovirally infected with mouse Myc using pMSCV-GFP-Myc plasmid. We used the same infection protocol as above and the virus was collected in serum free HGM medium. Serum was added to the plate the day before the cells needed to be split. Once the cells were immortalized, they were cultivated in 10% serum DMEM.

### Experimental animals

Manipulated cells were injected subcutaneously or into the spleen. Nude mice were gamma irradiated (400 rad) the day before injections. To generate subcutaneous tumors, we used 5 weeks old female nude mice and injected 10^6^ cells (unless otherwise noted in the figure legend) resuspended in 200 μl MEM. Tumor volume (cm^3^) was calculated as 0.52 x length x width^2^. Intrasplenic injections were done modifying a previously described method using 10-week-old female mice. Briefly, C57BL/6 mice received 2 doses of retrorsine 70 mg/kg i.p. before injection[36]. To avoid cell leakage into the mouse body, lower pole of the spleen was taking outside of the peritoneum to inject 2x10^6^ cells (unless otherwise noted in the figure legend) resuspended in 100 μl MEM medium. After injection, the lower pole of the spleen was tightly ligated with a 4–0 vicryl ligation. C57BL/6 mice received 3 doses of 0.5 ml/kg i.p. CCl4 every 5 days after transplantation. Intrasplenic injected nude mice were neither treated with retrorsine or CCl4. Tumor progression was monitored using whole body palpation and GFP imaging.

Colony formation assay

1000 cells were plated in triplicate on 6-well plates. Medium was changed every 3 days. After 3 weeks culture, cells were methanol fixed and stained with 0.5% crystal violet. After pictures were taken, crystal violet was dissolved with 0.1% SDS over night. Dissolved crystal violet staining was read at 595 nm using the Victor 3 machine (Perkin Elmer).

Immunoblotting

Immunoblotting was carried out using antibodies against RXRα (4RX3A2 kindly obtained from Dr Rochette-Egly, IGBMC 1:500 dilution ^25^), KRT18 (abcam ab32118, 1:10 000 dilution), KRT8 (abcam ab59400, 1:1000 dilution) and actin (abcam ab1801, 1:1000 dilution) or Ras Gap (BD Bioscience 610040, 1:1000dilution) as loading control. Briefly, proteins were separated by electrophoresis in SDS-polyacrylamide gel (10% acrylamide) and transferred to nitrocellulose electrophoretically at 110 V for 1 h 30 min. The nitrocellulose sheets were first blocked at room temperature for 1 hour in TBS containing 0.1% Tween and 5% nonfat dry milk. They were then sequentially incubated in primary and secondary antibodies diluted in blocking buffer. We used the LI-COR Odyssey infrared image system to visualize and quantify protein expression.

Immunohistochemistry

Fresh tissues were fixed in 10% formalin and paraffin embedded. Hematoxylin and eosin (H&E) sections were stained according to standard protocols. Immunohistochemistry was carried out using antibodies directed against PCNA (Abcam, 1:4000 dilution) and GFP (1:800 dilution). To block endogeneous peroxidase, 150 μm sections were treated with TBS containing 3% H_2_O_2_ for 15 min. After washing with TBS, antigen was retrieved in 0.1 M sodium citrate, for 5 min in microware, 40% power. DNase I treatment at 5 U/ml was used to enhance detection of nuclear proteins. Sections were treated with blocking buffer (TBS + 0.5% Tween20 + 1% BSA + 5% serum) for 1 h at room temperature and then incubated with primary antibodies diluted in blocking buffer overnight at 4°C. Binding of the antibodies was detected using biotin-conjugated anti-mouse or rabbit IgG diluted in blocking buffer and HRP-conjugated avidin diluted in TBS (Vectastain ABC kit, Vector) for 1 h and for 45 min respectively. Color development was achieved by treatment with the chromogen DAB (Vector Laboratories, Redwood City, CA) and was carried out for 5–10 min under a microscope. The slides were rinsed in tap water, counter-stained with hematoxylin.

## Supporting Information

S1 Fig Unsupervised clustering of RNA expression of normal and tumor mouse liver samples. The mouse tumors that originate from hepatoblasts[17,18] segregate with normal fetal liver isolated from E14.5, a developmental time at which livers are mostly hepatoblasts. The mouse tumors that originate from hepatocytes[19] segregate with post-natal liver samples as well as fetal liver isolated from E18.5, after the point at which hepatoblasts have differentiated into immature hepatocytes. See S3 Table for sample details.
(TIF)

S2 Fig Clustering of normal and tumor mouse liver samples based on expression of RXR-α and Wnt pathway genes. The mouse tumors that originate from hepatoblasts[17,18] segregate with normal fetal liver isolated from E14.5, a developmental time at which livers are mostly hepatoblasts. The mouse tumors that originate from hepatocytes[19] segregate with post-natal liver samples as well as fetal liver isolated from E18.5, after the point at which hepatoblasts have differentiated into immature hepatocytes. See S3 Table for sample details.
(TIF)

S3 Fig Clustering of normal fetal liver samples and normal adult liver samples based on expression of RXR-α and Wnt pathway genes. The fetal liver samples were taken from several different periods of embryonic development (E), and the adult liver samples were taken at various time points after partial hepatecomy (PH) [31].
(TIFF)

S4 Fig Classification of human HCC based on expression of RXR-α and Wnt pathway genes. HCC and non-malignant liver samples in the Wurmbach et al. dataset[25] were clustered based on expression of 138 RXR-α and Wnt pathway genes. Beneath the heatmap are three rows, showing for each sample (1) the one of two major clusters it belonged to following unsupervised clustering based on all genes; (2) relative prognosis based on the 65-gene signature of Kim et al., red = poor, green = good, white = neutral; (3) *CTNNB1*-mutation signature status, red = expression, green = less expression of the 5-gene signature associated with *CTNNB1* mutation[12].
(TIF)

S5 Fig Classification of human HCC based on expression of RXR-α and Wnt pathway genes. HCC samples in the Kim et al. dataset[24] were clustered based on expression of 138 RXR-α and Wnt pathway genes. Beneath the heatmap are three rows, showing for each HCC sample (1) the one of two major clusters it belonged to following unsupervised clustering based on all genes; (2) relative prognosis based on the 65-gene signature of Kim et al.[24], red = poor, green = good, white = neutral; (3) *CTNNB1*-mutation signature status, red = expression, green = less expression of the 5-gene signature associated with *CTNNB1* mutation[12].
(TIF)

S1 Table Focal amplicons observed in transpantable hepatoblast-derived tumors. (XLSX)

S2 Table List of genes in the RXR- α and Wnt- pathways (XLSX)

S3 Table List of mouse tumors and normal liver samples used for gene expression analysis (XLSX)
