# Supplementary figures and images for: Reciprocal Interaction of Wnt and RXR-α Pathways in Hepatocyte Development and Hepatocellular Carcinoma
Source: PLoS One. 2015 Mar 4;10(3):e0118480. doi: 10.1371/journal.pone.0118480 (PMC4349704; doi:10.1371/journal.pone.0118480)

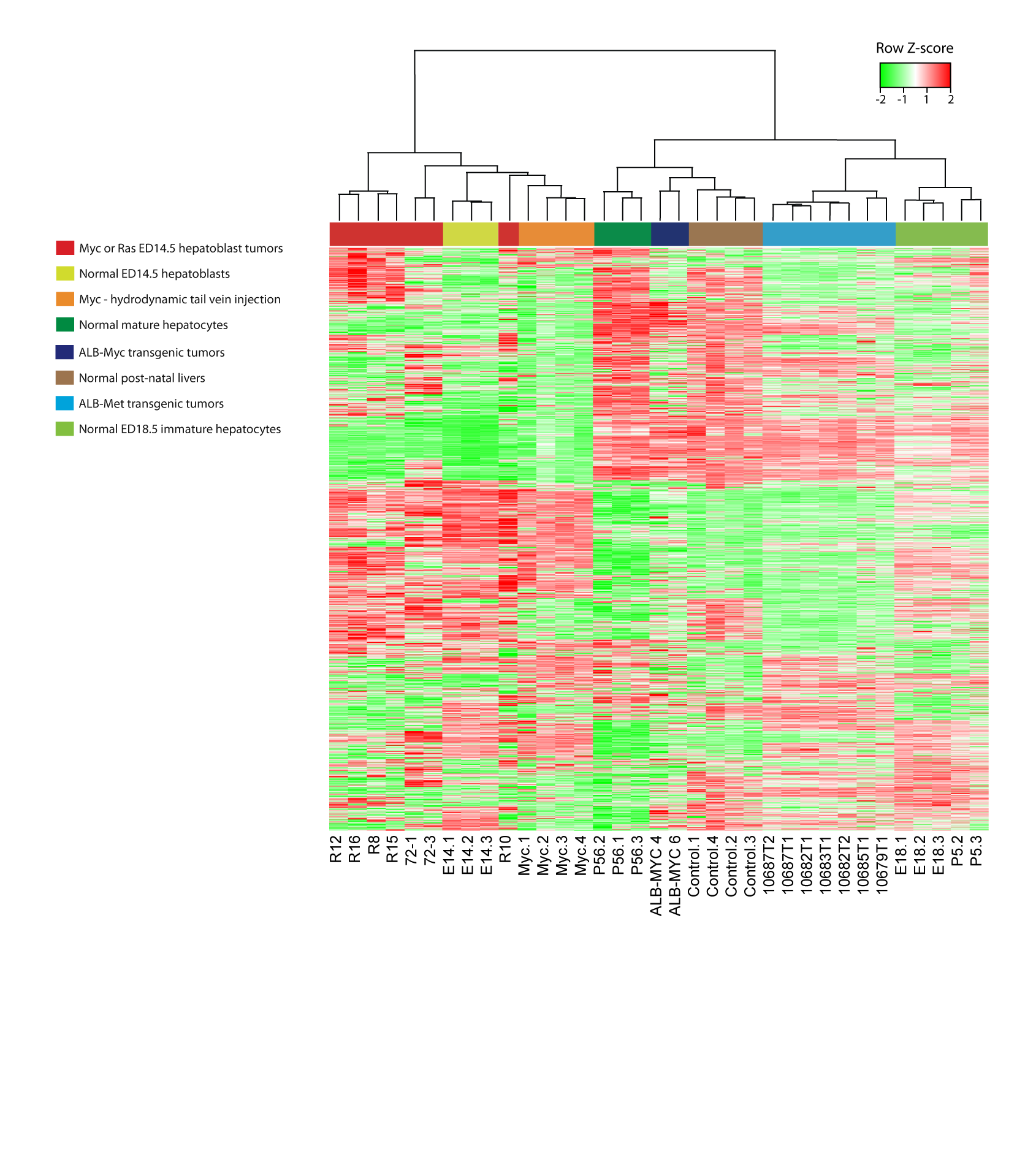

Supplement: S1 Fig — The mouse tumors that originate from hepatoblasts[17,18] segregate with normal fetal liver isolated from E14.5, a developmental time at which livers are mostly hepatoblasts. The mouse tumors that originate from hepatocytes[19] segregate with post-natal liver samples as well as fetal liver isolated from E18.5, after the point at which hepatoblasts have differentiated into immature hepatocytes. See S3 Table for sample details. (TIF) [file pone.0118480.s001.tif]

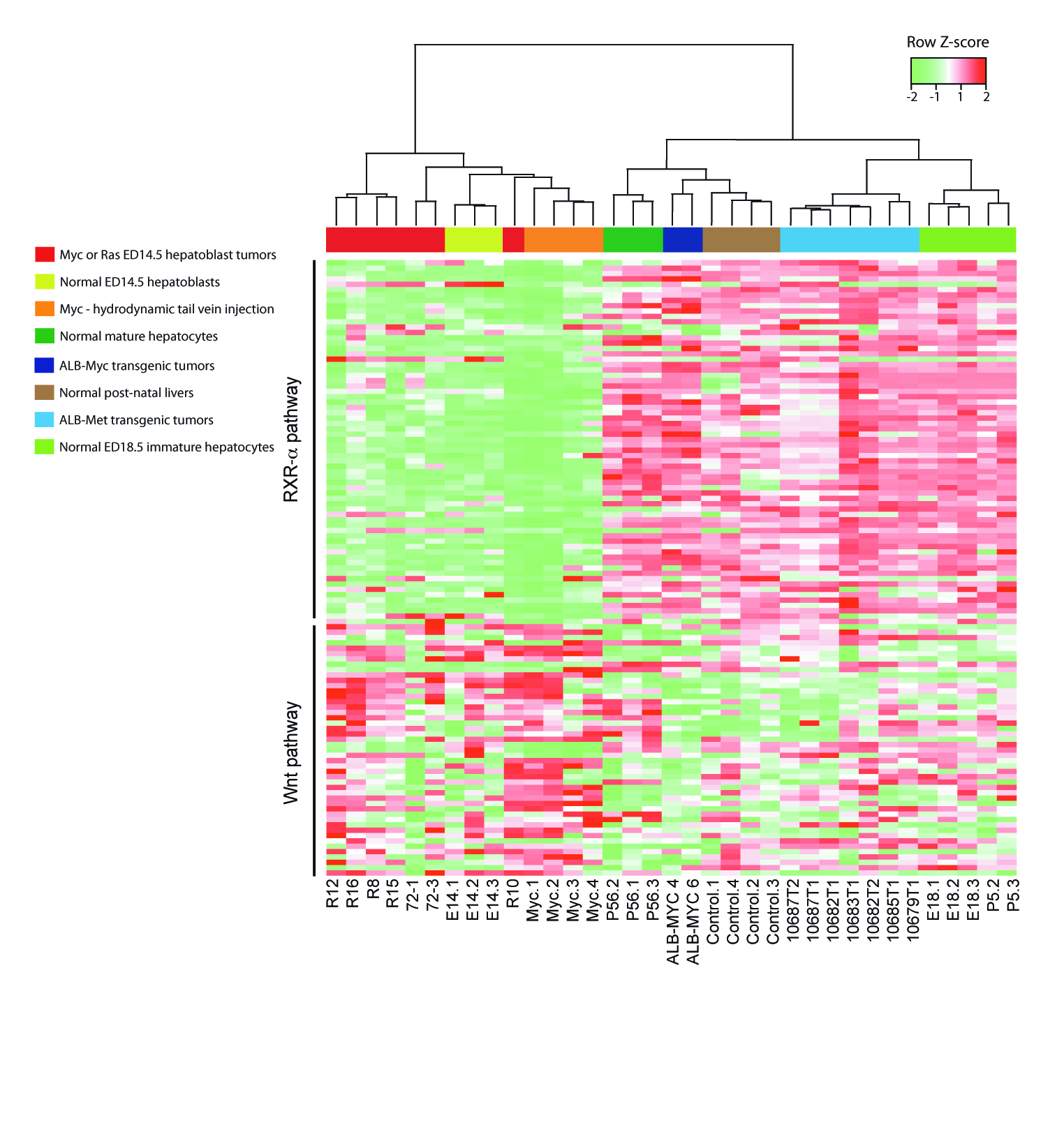

Supplement: S2 Fig — The mouse tumors that originate from hepatoblasts[17,18] segregate with normal fetal liver isolated from E14.5, a developmental time at which livers are mostly hepatoblasts. The mouse tumors that originate from hepatocytes[19] segregate with post-natal liver samples as well as fetal liver isolated from E18.5, after the point at which hepatoblasts have differentiated into immature hepatocytes. See S3 Table for sample details. (TIF) [file pone.0118480.s002.tif]

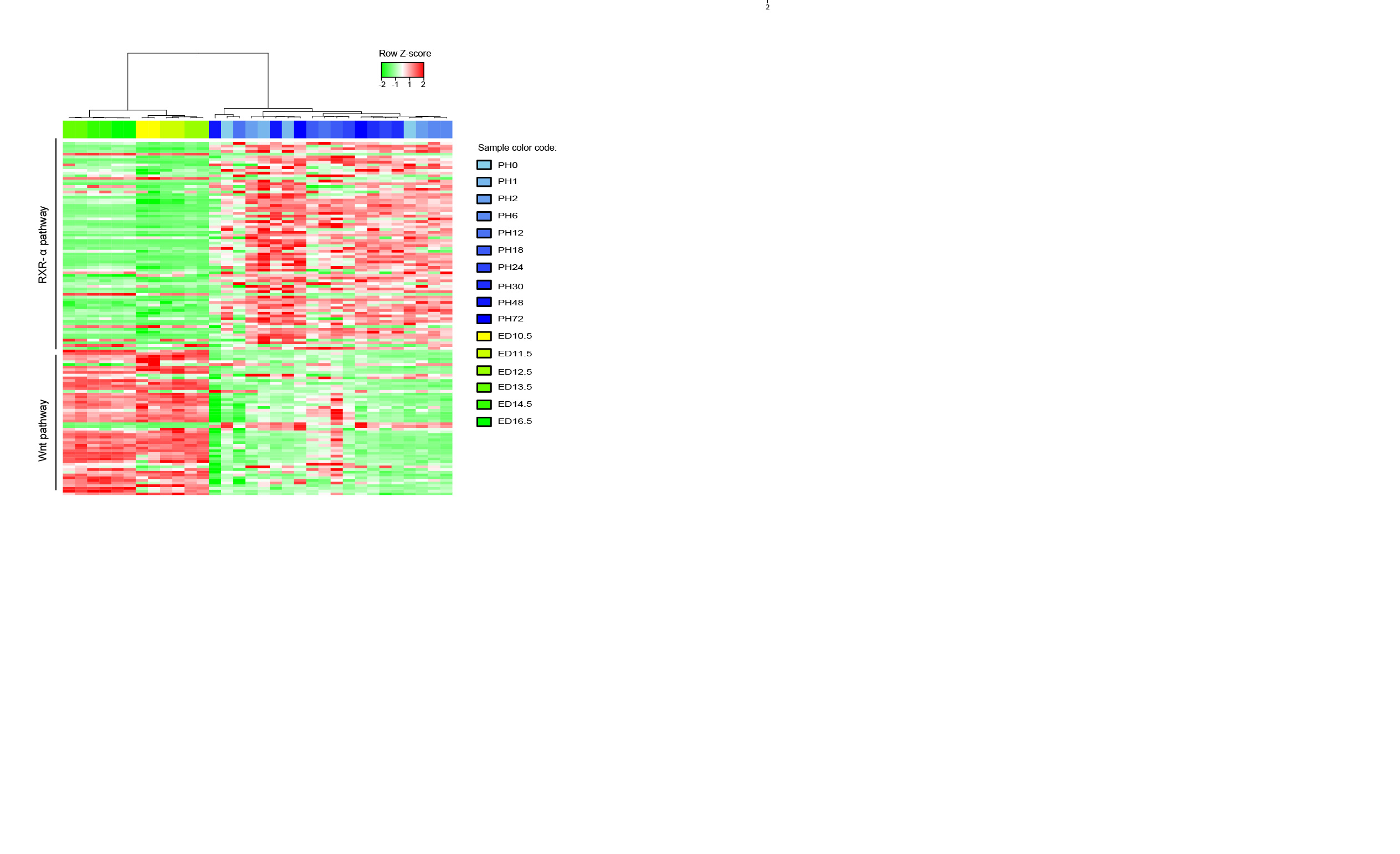

Supplement: S3 Fig — The fetal liver samples were taken from several different periods of embryonic development (E), and the adult liver samples were taken at various time points after partial hepatecomy (PH) [31]. (TIFF) [file pone.0118480.s003.tiff]

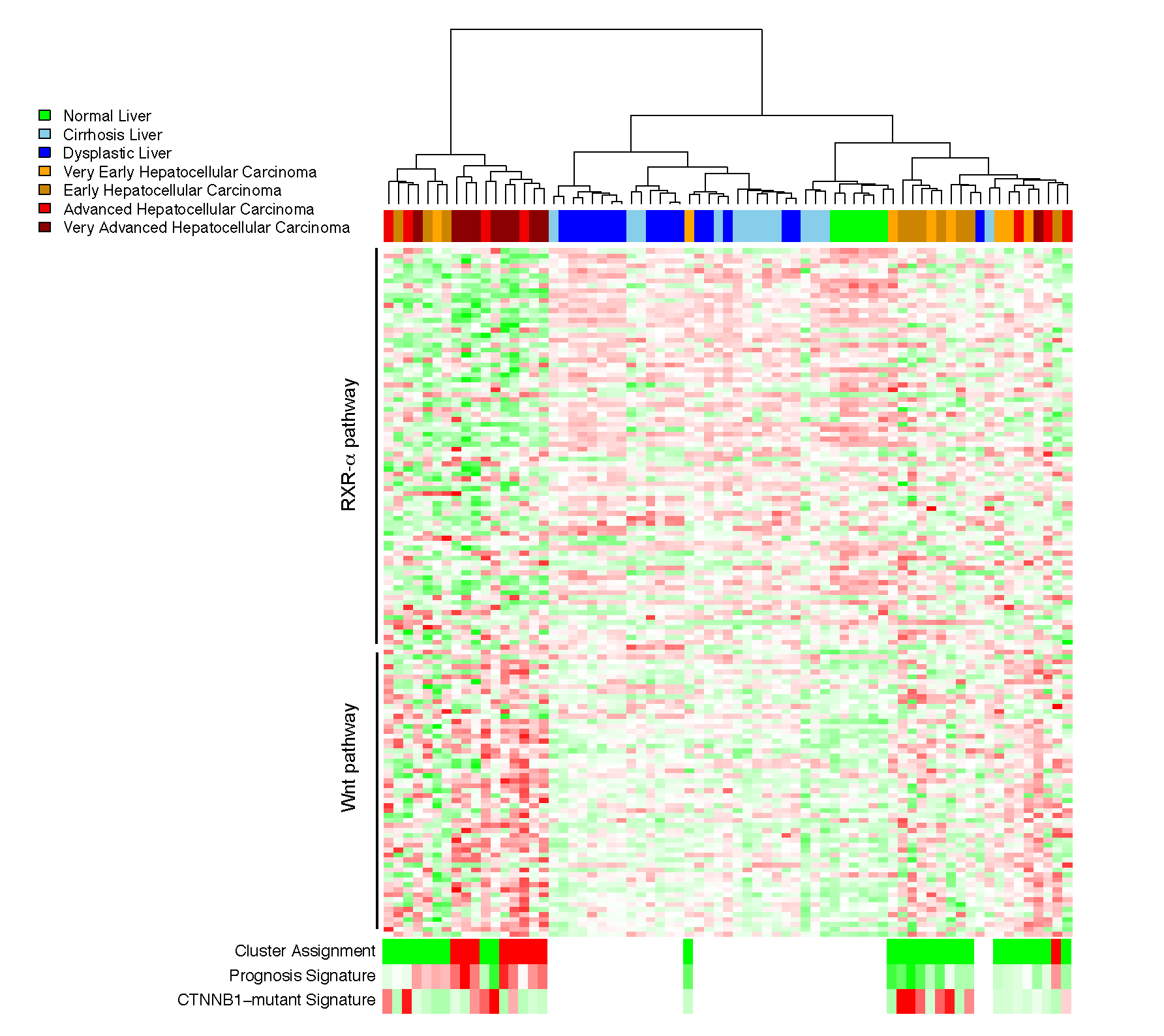

Supplement: S4 Fig — HCC and non-malignant liver samples in the Wurmbach et al. dataset[25] were clustered based on expression of 138 RXR-α and Wnt pathway genes. Beneath the heatmap are three rows, showing for each sample (1) the one of two major clusters it belonged to following unsupervised clustering based on all genes; (2) relative prognosis based on the 65-gene signature of Kim et al., red = poor, green = good, white = neutral; (3) CTNNB1-mutation signature status, red = expression, green = less expression of the 5-gene signature associated with CTNNB1 mutation[12]. (TIF) [file pone.0118480.s004.tif]

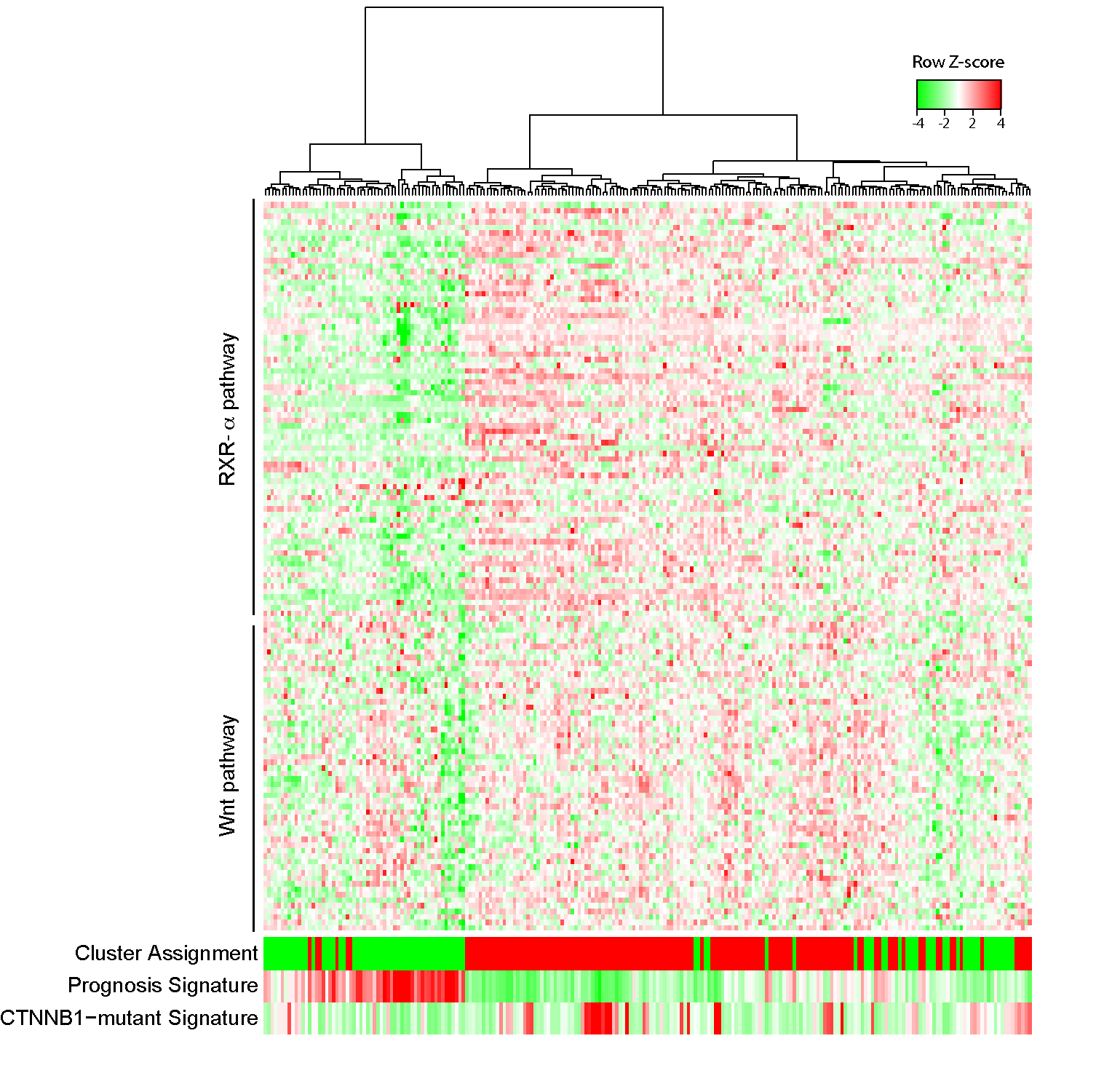

Supplement: S5 Fig — HCC samples in the Kim et al. dataset[24] were clustered based on expression of 138 RXR-α and Wnt pathway genes. Beneath the heatmap are three rows, showing for each HCC sample (1) the one of two major clusters it belonged to following unsupervised clustering based on all genes; (2) relative prognosis based on the 65-gene signature of Kim et al.[24], red = poor, green = good, white = neutral; (3) CTNNB1-mutation signature status, red = expression, green = less expression of the 5-gene signature associated with CTNNB1 mutation[12]. (TIF) [file pone.0118480.s005.tif]
